# The Potential Role of Coagulation Factor Xa in the Pathophysiology of COVID-19: A Role for Anticoagulants as Multimodal Therapeutic Agents

**DOI:** 10.1055/s-0040-1718415

**Published:** 2020-10-07

**Authors:** Galit H. Frydman, Michael B. Streiff, Jean M. Connors, Gregory Piazza

**Affiliations:** 1 Coagulo Medical Technologies, Inc., Auburndale, Massachusetts, United States; 2 Center for Biomedical Engineering, Department of Biological Engineering, Massachusetts Institute of Technology, Cambridge, Massachusetts, United States; 3 Division of Trauma, Emergency Surgery and Surgical Critical Care, Department of Surgery, Massachusetts General Hospital, Boston, Massachusetts, United States; 4 Division of Hematology, Department of Medicine, The Johns Hopkins University School of Medicine, Baltimore, Maryland, United States; 5 Division of Hematology, Department of Medicine, Brigham and Women's Hospital and Harvard Medical School, Boston, Massachusetts, United States; 6 Division of Cardiovascular Medicine Department of Medicine, Brigham and Women's Hospital, Boston, Massachusetts, United States

**Keywords:** SARS-CoV-2, coronavirus, COVID-19, coagulation, factor X, factor Xa, anticoagulants

## Abstract

SARS-CoV-2 infection (COVID-19) results in local and systemic activation of inflammation and coagulation. In this review article, we will discuss the potential role of coagulation factor Xa (FXa) in the pathophysiology of COVID-19. FXa, a serine protease, has been shown to play a role in the cleavage of SARS-CoV-1 spike protein (SP), with the inhibition of FXa resulting in the inhibition of viral infectivity. FX is known to be primarily produced in the liver, but it is also expressed by multiple cells types, including alveolar epithelium, cardiac myocytes, and macrophages. Considering that patients with preexisting conditions, including cardiopulmonary disease, are at an increased risk of severe COVID-19, we discuss the potential role of increased levels of FX in these patients, resulting in a potential increased propensity to have a higher infectious rate and viral load, increased activation of coagulation and inflammation, and development of fibrosis. With these observations in mind, we postulate as to the potential therapeutic role of FXa inhibitors as a prophylactic and therapeutic treatment for high-risk patients with COVID-19.

## Introduction

Coronaviruses are small, enveloped, positive-sense single-stranded RNA viruses which can infect multiple species. A unique group of beta-coronaviruses have caused severe disease in humans over the past few decades, including severe acute respiratory syndrome (SARS)-CoV, Middle East respiratory syndrome (MERS), and, most recently, SARS-CoV-2 (COVID-19). 1 Coronaviruses are very common and clinical symptoms can range from asymptomatic and mild (“cold” symptoms) to moderate to severe symptoms, including fever, upper and lower respiratory tract infection, gastrointestinal symptoms, and even the progression into systemic inflammatory response syndrome (SIRS), acute respiratory distress syndrome (ARDS), and multi-organ dysfunction syndrome (MODS). 2 3 4 COVID-19 was first detected in Wuhan, China, in December 2019. It has since rapidly spread throughout the world, with a very high infectivity rate but with mixed clinical symptoms. 5 The cause of the wide range of clinical symptoms is likely multifactorial, including route of exposure and infectious dose, baseline health status of the patient, and potentially differences between the various strains of SARS-CoV-2 that continue to develop over time. 6 7 While many patients who have been diagnosed with COVID-19 are asymptomatic or have mild signs, such as a cough or sore throat, men, patients ≥ 60 years old, and those with preexisting conditions, such as obesity, pulmonary disease, cardiovascular disease, or diabetes, have been subject to much more severe symptoms and a higher mortality rate. 8 9 10 11 12 13 In this review article, we will discuss the known and proposed mechanisms by which COVID-19 successfully infects host cells, how this mechanism leads to the resulting pathophysiology of the disease, and potential therapeutic approaches to reduce these pathologic effects, all with a focus on the potential role of coagulation factor X (FX).

## Coronavirus Mechanism of Infection and Cellular Tropism

To evaluate the potential role of FX in the pathogenesis of SARS-CoV-2 infection, we must first review the basic biology of the virus and its cellular tropism that leads to its localization to specific organ systems. Coronaviruses are composed of RNA and proteins internally and then a nuclear envelope with spike glycoproteins. The SPs are peplomers that determine the cellular host tropism. SP contains a type II fusion machine (spike protein 2 [S2]) and a receptor binding domain (RBD) on spike protein 1 (S1). 14 15 For both SARS and COVID-19, one of the primary host cells receptors that the SP binds to is angiotensin-converting enzyme 2 (ACE2). 16 17 18 19 ACE2 plays a primary role in the renin–angiotensin system (RAS) and, more specifically, reduces angiotensin II (ATII) levels which are associated with increased inflammation, apoptosis, fibrosis, and oxidative stress. 20 21 Binding of the SP to ACE2 has been shown to reduce cellular ACE2 expression, induced by viral shedding of the receptor, and increase inflammatory cytokine production, such as tumor necrosis factor (TNF)-α. Increased levels of ATII, such as with the blocking or reduction of ACE2, has been shown to play a role in the pathophysiology of ARDS as well as diabetes. 16 17 18 19 20 21

Once the SP is bound to ACE2, the SP is subject to proteolytic cleavage into S1 and S2. 20 One of the most well-known proteolytic enzymes that performs SP cleavage is transmembrane protease, serine 2 (TMPRSS2). 22 23 24 TMPRSS2 colocalizes with ACE2 on multiple cell types, including type II pneumocytes and cardiac myocytes and is thought to play a key role in the infection of the pulmonary and cardiovascular system. 8 25 26 27 28 29 Once cleaved, S1 is released (along with ACE2) and S2 remains attached to the host cell, playing a role in cell–virus cellular membrane fusion. Membrane fusion is the first step in the active infection of the cell, in which the genetic material and proteins from the virus are inserted into the host cell and replicate. The new virions are then released, commonly causing cellular stress and cell death, thus further promoting inflammation while increasing the body's viral load. 14 15 30

Based on the mechanism of infection, it is not surprising that patients with comorbidities, such as heart disease, pulmonary disease, and diabetes, are at increased risk due to potential baseline changes in ACE2 expression, changes in RAS system activation, and preexisting pulmonary or cardiac injury with fibrosis, inflammation, or both. In these cases, it is thought that the virus localizes within these already-compromised organ systems and, through viral infection, increases local inflammation and oxidative and cellular stress resulting in pulmonary and cardiovascular damage. 3 8 24 31

## Coagulation Factor X and Coronavirus Spike Protein

While TMPRSS2 has been shown to be one of the primary proteolytic enzymes that cleaves coronavirus SP, this cleavage can be accomplished via multiple other enzymes which bind to the SP. Studies with SARS virus have demonstrated that coagulation factor Xa (FXa) is capable of cleaving SARS SP into S1 and S2 in a dose-dependent manner. The addition of Ben-HCl, a protease and FXa inhibitor (FXai), was shown to inhibit SP cleavage by FXa and the addition of Ben-HCl also inhibited in vitro cell infectivity. 32 Interestingly, these findings are consistent with various studies demonstrating that heparin treatment has antiviral activity in in vitro experiments with CoV, influenza, metapneumovirus, human immunodeficiency virus (HIV), respiratory syncytial virus (RSV), and hepatitis. 33 34 35 36 37 Similarly, additional studies have demonstrated the role of various coagulation factors, including FXa, factor IIa (FIIa; thrombin), and plasmin, as proteases, which act upon SARS SP. 38

**Fig. 1:**
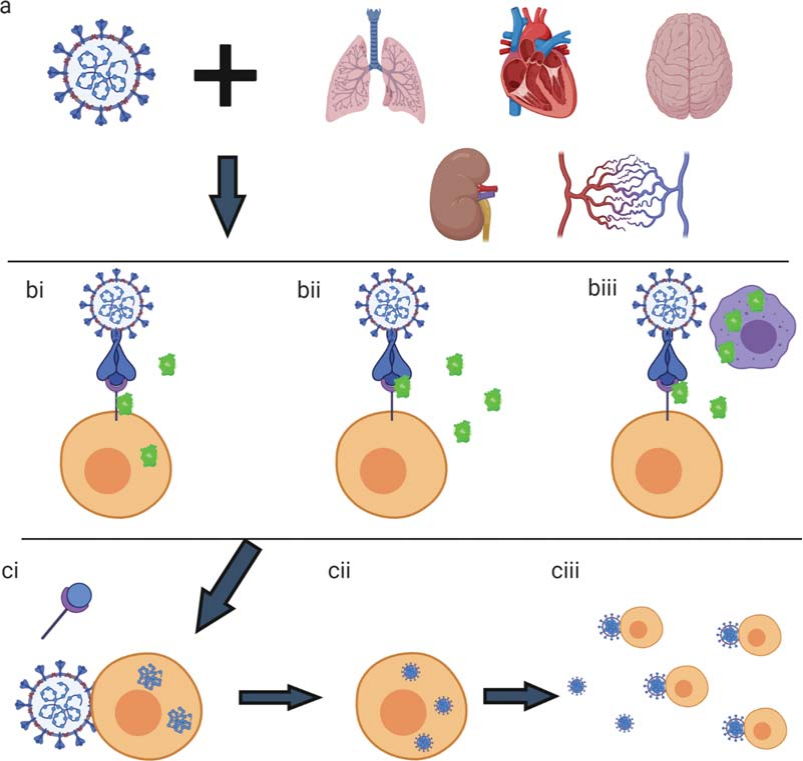
The potential role of factor Xa in Co-V cellular infection. Top: The coronavirus (left) binds to multiple cells that express receptors that bind to the coronavirus spike protein (a). For example, the spike protein can bind to ACE2 receptors, which are present in the alveolar and bronchiolar epithelium, cardiac myocytes, the brain/central nervous system, the kidneys (primarily the proximal tubules), and the vascular endothelium. Middle: Once the spike protein is bound to the host cell receptor, a proteolytic enzyme binds to the spike protein to cleave the protein into spike protein 1 and spike protein 2. In this example, the proteolytic enzyme is serine protease, factor Xa. (bi) Factor X and factor Xa can be expressed by the host cell directly, allowing for colocalization of the spike protein receptor and the serine protease. (bii) Factor Xa can be present, unbound in the circulation. (biii) Factor X and factor Xa can be localized to the spike protein by nearby cells expressing factor X and factor Xa, such as macrophages. Bottom: Once the spike protein is successfully cleaved by the proteolytic enzyme, spike protein 1 is released with or without the bound host cell receptor, while spike protein 2 aids in the fusion of the viral and host cell membrane. (ci) The virus and host cell membrane are fused and the viral genetic material is inserted into the host cell. (cii) The viral genetic material replicates within the host cell. (ciii) New coronavirus viral particles are released by the host cell, resulting in infection of new host cells as well as propagation of inflammation and coagulation.

FX is a coagulation factor and serine protease, which is vitamin K dependent and primarily synthesized by the liver. 39 Interestingly, FX has been shown to be expressed in other cells types, including alveolar and bronchiolar epithelium, cardiac myocytes, and brain tissue, cells that also happen to express ACE2. 29 40 41 42 43 44 45 46 While TMPRSS2 was thought to be the primary protease responsible for the cleavage of COVID-19 SP due to its colocalization with ACE2 on host cells, it is likely that FX expression (along with other coagulation factors such as FIIa) by these cells may also serve as a localized protease which can cleave SP upon host cell receptor binding ( Fig. 1 ).

## Factor X and Its Potential Role in the Pathophysiology of Coronavirus Infection

**Fig. 2:**
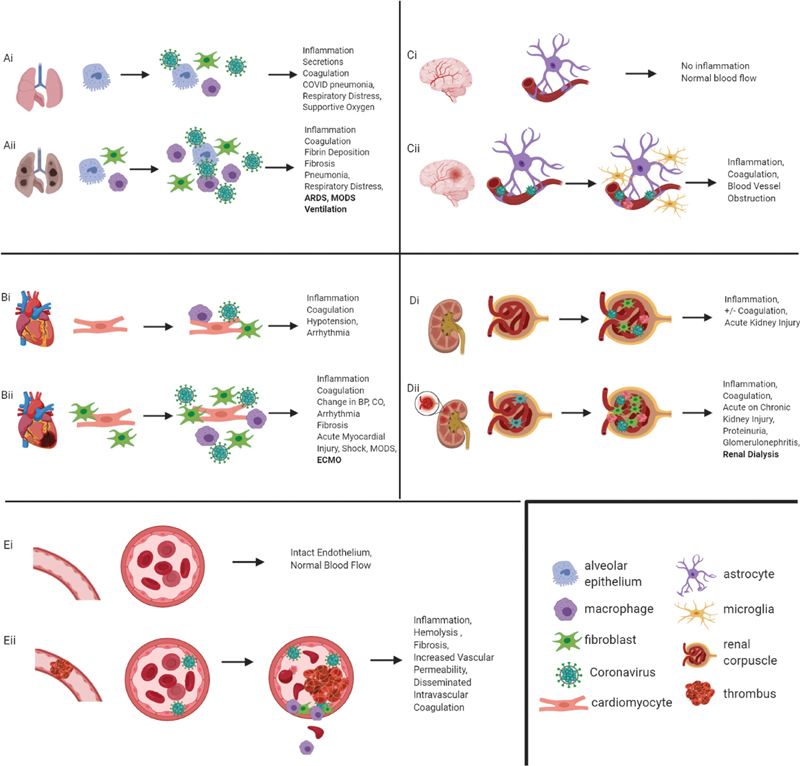
The potential role of factor Xa in the pathophysiology of COVID-19. This figure represents the potential sequelae of COVID-19 in the context of various organ systems that are known to express factor Xa (FXa). ( **A** ) demonstrates the consequences of COVID-19 in both healthy (Ai) and diseased (Aii) lungs. In the diseased lungs, the presence of preexisting inflammation and fibrosis exacerbates further inflammation and fibrosis. ( **B** ) demonstrates the consequences of COVID-19 in both the healthy (Bi) and diseased (Bii) heart. Similar to the pulmonary system, the presence of preexisting fibrosis further exacerbates the inflammation and fibrosis secondary to COVID-19. ( **C** ) represents both uninfected brain tissue (Ci) and brain tissue with COVID-19 (Cii). The infected tissue demonstrates an increase in microglial activation, inflammation, and intravascular thrombosis. Panel D represents the consequences of COVID-19 in both health (Di) and diseased (Dii) kidneys (specifically focusing on the glomerulus). The infected tissue demonstrates increased inflammation, fibrosis, and capillary thrombosis. Panel E represents blood vessels and the role of the vascular endothelium in COVID-19. The healthy, uninfected blood vessel (Ei) has an open lumen with laminar blood flow, while the infected blood vessel (Eii) has endothelial damage resulting in increased inflammation, fibrosis, vascular permeability, thrombosis formation, and turbulent flow resulting in damage to red blood cells in the form of hemolysis and schistocyte formation.

FX and FXa play a role in the pathophysiology and progression of various forms of cardiopulmonary disease. Although FX/FXa is most commonly known to be present in soluble form within the circulatory system, it is also expressed by multiple cell types, including alveolar and bronchiolar epithelium. 41 In the alveolar and bronchiolar epithelium, the presence of reactive oxygen species (ROS) increases the expression of FX. 41 42 FXa has also been shown to be a potent inducer of lung fibrosis via transforming growth factor-β (TGF-β), mediated by proteinase-activated receptor-1 (PAR1). 42 FXa is locally expressed within the lung and has been shown to be one of the factors driving the fibrotic response to lung injury. 42 47 48 Considering that patients with preexisting pulmonary and cardiac disease appear to be among the highest risk group for severe COVID-19, it is possible that these patients may have a higher baseline expression of FX in these cell populations, putting them at increased risk of host cell infection, with factor X serving as one of the cleavage proteins for SARS-CoV-2 ( Fig. 2A ).

FX is known to be expressed by cardiac myocytes and fibroblasts and has been found to be expressed in the heart following pressure overload. In a recent study, rivaroxaban, a FXai, was demonstrated to reduce inflammation, hypertrophy, and fibrosis secondary to pressure overload and improve diastolic function, even at subtherapeutic doses (e.g., doses that did not affect thrombin generation), and was shown to correlate with decrease expression of FX in these cell populations. 43 In light of the fact that cardiac myocytes are known to express ACE2 and FX, at increased levels in cardiac disease, this poses another probable mechanism by which COVID-19 directly infects cardiac cells, and potentially at an increased level in patients with preexisting disease ( Fig. 2B ). 49 50 51

In addition to significant effects on the cardiopulmonary system, SARS-CoV-2 infection has also been shown to be associated with pathological effects on the brain and the kidneys. 52 53 54 55 COVID-19 has been observed to have primary effects in the brain with patients presenting with confusion, abnormal behavior, seizures, and brain swelling. 53 Not surprisingly, FX was found to be expressed in the rat brain and central nervous system, which is also known to contain an abundance of tissue factor as well. 44 It is already known that ACE2 is present within the brain, and it is therefore plausible that this is another potential site of FX/ACE2 colocalization allowing for COVID-19 binding and infection, with subsequent inflammation and potential localized activation of coagulation ( Fig. 2C ). 45

As with other organ systems involved in MODS in critically ill patients, the development of microthrombi during the systemic inflammatory response is a likely culprit for the development of acute kidney injury during COVID-19. 52 54 55 56 However, FX also may have its own role in the development and progression of kidney injury ( Fig. 2D ). For example, the interaction between FXa and protease-activated receptor 2 (PAR2) has been shown to play a role in nephritis and glomerular nephritis in animal models. FXa inhibitors, such as fondaparinux, have been shown to suppress the development of proteinuria, glomerular hypertrophy, and angiogenesis in diabetic mice and decrease the thickness of fibrotic tissue and angiogenesis in a model of peritoneal fibrosis. 57 58 The glomeruli itself has a glomerular procoagulant activity (PCA) that has been under investigation, with animal studies demonstrating that PCA includes a unique serine protease that directly activates FX. 59 Additionally, in mouse models of lupus nephritis, this unique FX activator was bound to dense deposits, macrophages, and endothelial cells of diseased glomeruli. 59 This finding is especially interesting considering that an increasing number of clinical studies have also demonstrated increased levels of lupus autoantibodies in patients with COVID-19, although these may be two very different mechanisms of inflammation and coagulation derangement. 60 61 62

Recently, the role of the vascular endothelium in the pathology of COVID-19 has been reported to potentially be a primary driver in the development of intravascular thrombosis and related sequelae. 63 64 65 66 67 Although it is beyond the scope of this article to discuss the very important and vast role of the endothelium in inflammation and coagulation, we do want to touch upon the potential role of FX in this paradigm ( Fig. 2E ). Although endothelial cells are currently most well-known for the activation of prothrombin via exogenous FXa, they have been shown (in vitro) to produce a variety of coagulation factor themselves, including factors VII, IX, X, and tissue factor. 68 69 70 While the endothelium does express its own coagulation factors, it also is capable of expressing anticoagulant factors as well, such as tissue factor pathway inhibitor (TFPI), which serve a protective role. 71

## Factor X and Its Potential Role in Coronavirus-Related Inflammation

Coronavirus disease, especially SARS and COVID-19, frequently results in increased plasma inflammatory cytokines, and evaluation of pulmonary pathology has revealed an increase in macrophage and lymphocyte infiltration with fibrin deposition. 72 73 Patients suffering from severe coronavirus infection have also been shown to suffer from various states of coagulopathy and systemic inflammation. 13 74 A recent article by Masi et al performed a comprehensive evaluation of multiple coagulation-related biomarkers and identified that among the coagulation factors, VII, VIII, II, V, and X were significantly elevated in patients with COVID-19-related ARDS. 52 Macrophages are one of the primary inflammatory cells involved in the response to coronavirus-related immune response. 75 Some SARS studies have demonstrated that SARS-CoV is capable of replicating in human peripheral monocytes and macrophages as well as in alveolar macrophages. 76 77 Interestingly, alveolar macrophages have been demonstrated to express FX and FXa, in addition to other coagulation factors, such as factor VII (FVII), which would also provide another route of FX activation. 78 79 80 81 In fact, one study demonstrated that monocytes and macrophages were a crucial source of extravascular FX in the tumor microenvironment promoting tumor immune evasion, with rivaroxaban treatment improving antitumor immunity. 82 In addition to their role in inflammation and coagulation, localized macrophages may provide an additional source of FXa to nearby cells expressing the ACE2 receptor and bound to coronavirus; this would result in an additional route of SP cleavage and increased cell infection.

FXa also plays a major role in FIIa-mediated PAR activation. FXa is upstream of prothrombin (FII) and is capable of activating FII to FIIa, which cleaves PAR1, PAR3, and PAR4, resulting in cellular activation. PAR2 is primarily activated by FXa. PARs are expressed on platelets, leukocytes, and endothelial cells. PAR1 plays a critical role in inflammation and is present on endothelial cells and fibroblasts. When activated, PAR1 stimulates the production of monocyte chemoattractant protein-1 (MCP1), TNF-α, IL1B, IL6, and TGF-β. This PAR1 activation also activates cells, resulting in P- and E-selectin exposure. 83 One can imagine that in the case of a patient with preexisting lung disease, such as idiopathic fibrosis or asthma, where there is already an increase in FX expression and activation, along with increased PAR1 activation and fibroblasts, and that this would create a naturally hospitable environment for the binding of SARS-CoV-2, increasing cell infection and the development of severe lung pathology, such as ARDS ( Fig. 2A ). As mentioned in the earlier section, PAR1 and PAR2 have both been shown to interact with FX/FXa and play a role in kidney-related inflammation and thrombosis ( Fig. 2D ). 57 58 Additionally, the classic concept of inflammation begetting thrombosis and vice versa is nicely illustrated by the observation that PAR1 plays a critical role in thrombin-mediated platelet activation.

## Factor Xa Inhibitors as a Potential Treatment for COVID-19

In this review, we provide support based on previous literature that FX and FXa may play a role in the infection and clinical symptoms of COVID-19 patients. We propose that FX may in fact be an additional colocalized protease on cells coexpressing ACE2 and may serve as a protease for SP cleavage. Additionally, the expression of FX/FXa by alveolar macrophages may provide an additional localized source of protease for the cleavage of SP. In this model of infectivity, there is a continuum of pathology, where patients with preexisting cardiopulmonary inflammatory disease may have increased baseline levels of ACE2 and FX/FXa, which increases SARS-CoV-2 binding and infection, leading to further cellular activation, inflammation, and coagulation which further perpetuates SARS-CoV-2 replication.

**Fig. 3:**
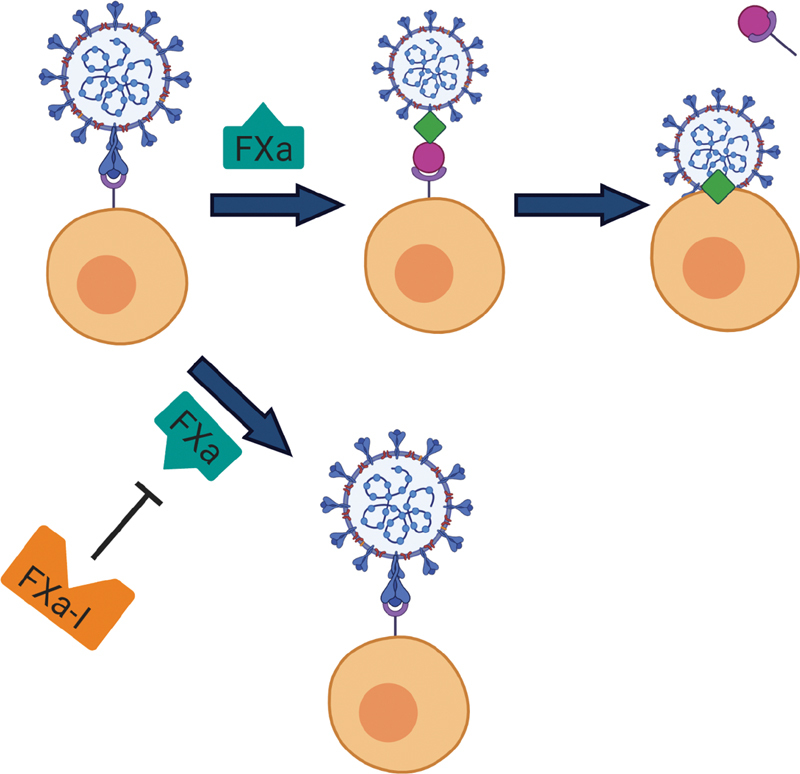
Proposed therapeutic mechanism of factor Xa inhibitor on CoV cellular infection. (Top) SARS coronavirus binds to the host cell expressing a receptor (purple) that binds to the SARS coronavirus spike protein (blue club-shape). Factor Xa (FXa) then acts as a proteolytic enzyme, cleaving spike protein into spike protein 1 (pink circle) and spike protein 2 (green diamond). The spike protein 1 then parts from the virus–cell complex with or without the attached receptor, while spike protein 2 serves to aid in the fusion of the virus and cell membranes. (Bottom) The addition of a factor Xa inhibitor (FXa) blocks FXa from acting as a proteolytic enzyme, therefore leaving the spike protein intact and preventing virus and host cell membrane fusion.

Based on this information, it is no surprise that critically ill COVID-19 patients have increased inflammatory cytokines and coagulopathies. Because FX/FXa may play a role in the pathophysiology of the COVID-19, factor Xa inhibitors represent a potential novel therapeutic modality ( Fig. 3 ). The inhibition of FXa could aid in the reduction of cell infectivity by reducing FXa-dependent proteolytic cleavage of SP, whether the FXa is localized on the host cell, released by nearby cells, or in the circulation. Additionally, FXai could also help control the inflammation and coagulation-related stimulation by SARS-CoV-2 infection, which may explain the clinical data suggesting the benefit of heparin administration in critical patients. 84 85 86 For example, oral FXai has been demonstrated to result in a dose-dependent decreases in inflammatory cytokines by LPS-activated monocytes. 86 This anti-inflammatory effect, in conjunction with its traditional anticoagulant effect, can be especially effective in the case of patients with increased baseline levels of inflammation and oxidative stress which are present with certain comorbidities, such as pulmonary fibrosis and asthma.

Because coagulation and inflammation are so intertwined, an increase in inflammation will likely first trigger an adaptive and protective hemostatic response—this can quickly turn into a hypercoagulable response, making it more likely for patients to develop pathologic thrombosis. 83 87 88 During this phase, an increase in fibrin deposition may obstruct alveolar spaces and micro capillaries. 89 There is also an increase in immunothrombotic complex formation, such as platelet–leukocyte aggregates, platelet activation, extracellular histone release, and neutrophil extracellular trap (NET) formation, which further promotes and exacerbate inflammation and coagulation. 89 90 91 These responses create a perfect storm for the obstruction of blood vessels and airways resulting in the development of MODS and ARDS in patients with severe inflammation. 89 92 93 Due to this propensity toward hypercoagulability, it is common for patients at risk of severe inflammation to receive prophylactic or therapeutic anticoagulant treatment, usually with unfractionated heparin (UFH) or low-molecular-weight heparin (LMWH) to prevent the development of venous thromboembolisms (VTEs). 94 95 In addition to the traditional intravenous and subcutaneous administration of heparin, inhaled UFH and LMWH have been proposed and used in the context of pulmonary inflammation and ARDS, although optimal dosing and formulation is still under investigation. 96 97 98 99

Poor prognosis is associated with COVID-19 patients who display systemic inflammation in conjunction with coagulopathy, as manifested by increased D-dimers and fibrin degradation products (FDPs). 74 It has also been demonstrated that critically ill COVID-19 patients treated with anticoagulants, including UFH and LMWH, have a better prognosis as shown by increased survival and decreased need for mechanical ventilation. 84 85 Because FXa has been shown to play an important role in both inflammation and coagulation, there may be a place for FXai in the treatment of both SARS-CoV-2-related coagulopathy and SARS-CoV-2-related inflammation and infection, especially in hospitalized patients with low risk of bleeding. Although injectable indirect FXai are common in the hospital setting, such as UFH and LMW, direct oral FXai, such as rivaroxaban, apixaban, and edoxaban, are easily administered in an outpatient and inpatient setting as a once-a-day or twice-a-day pill and may be efficacious in more stable patients. 100 When considering whether a FXa inhibitor would be a potential COVID-19 therapeutic, it is important to take into account the stage and severity of the disease. For example, the administration of an oral FXai may be more appropriate and efficacious in an outpatient setting, where a patient may be symptomatic but otherwise stable; whereas, a patient who requires hospitalization, who likely has more severe inflammation and coagulation derangement, may benefit from an injectable anticoagulant, such as heparin, which can also target multiple coagulation factors simultaneously (FXa and FIIa), has a short half-life, and is easily reversible with protamine should urgent invasive procedure be necessary, or direct thrombin inhibitors, such as argatroban and bivalirudin, such as in the case of heparin-induced thrombocytopenia, or heparin resistance secondary to decreased levels of AT. 83 84 101

Multiple clinical trials have been started for the treatment of coagulation-related pathology, including for the prevention of VTE, and other treatments aimed at ARDS pathology secondary to SARS-CoV-2 infection, including the administration of inhaled UFH with N-acetylcysteine (HOPE clinical trial), for the prevention of clot formation and the loosening of mucous secretions, and the administration of inhaled tissue plasminogen activator (tPA), for the breakdown of fibrin clots that are already formed and are obstructing the airways and vessels. 102 Although not being evaluated as an anticoagulant, hydroxychloroquine and azithromycin, another drug combination that is being explored for the treatment of COVID-19, have also been shown to have anticoagulant properties via alternative mechanisms. 103 104 105 106 107 108 In addition to being used as an antimalarial, hydroxychloroquine has also been used more as a treatment for systemic lupus erythematosus, which poses another potential mechanism by which this combination may have anticoagulant effects during COVID-19. 60 61 62 109

If FXai are confirmed to be effective in the reduction of SP cleavage and cell infectivity, the administration of FXai may also serve as an outpatient treatment for patients who have recently been infected, patients at high risk of exposure, and for patients in the high-risk category by reduction of FXa levels. This is quite speculative, but in this scenario the FXai would be multimodal in action, including VTE prevention, anti-inflammatory, and, potentially, antiviral effects. Recent retrospective studies evaluated whether the administration of anticoagulation and antiplatelet drugs had any protective effect on patients with COVID-19 and found conflicting results. 110 111 One study found no significant difference in outcomes, including mortality and mechanical ventilation; however, this study utilized a propensity-matched cohort, which limited the anticoagulation sample size to <150 patients and the authors do not clarify which anticoagulant was administered, if antiplatelets were given simultaneously, and there was no control for anticoagulation treatment regimen once admitted into the hospital. 110 The second study, also small with a patient population of 70, evaluated elderly patients with chronic heart disease that were on anticoagulants for at least 6 months prior to the diagnosis of COVID-19-related interstitial pneumonia and found that direct oral anticoagulants (DOACs), factor IIa and factor Xa inhibitors, appeared to be significantly protective. 111 Although this is an important study and a step in the right direction, the authors acknowledge that more thorough studies need to be performed.

### Limitations

While in this review we go through the potential mechanisms by which FX/FXa may be involved with the pathophysiology of COVID-19, to date, there are no mechanistic in vitro studies that have been published exploring this question. Currently, the majority of publications have been clinical observations and retrospective studies, including reporting of currently available laboratory coagulation assay results as well as clinical outcomes for patients on various anticoagulation regimens. There is one study that evaluated the concentration of various coagulation factors in COVID-19 and non-COVID-19-related ARDS which did identify that there was a significant difference (increase) in the amount of FX in the COVID-19 ARDS patients. 52 Due to the high infectious rate and strain on the medical and scientific community, it is not surprising that most research has been limited to observational studies. Additionally, anticoagulation protocols vary between institutions and countries and are constantly changing, making it difficult to perform controlled prospective studies. 112 Although most clinicians agree that anticoagulation therapy is likely warranted in hospitalized patients with COVID-19, the drug choice and dosing paradigm is quite variable. 113 114 115 116 Outpatient studies are even more difficult to perform due to the requirement for quarantining if COVID-19 positive and the lack of availability of accurate, rapid, point-of-care diagnostic tests.

There are retrospective studies that can be performed that may help in the evaluation of the potential protective effects of FXai on COVID-19 clinical outcomes, specifically in patients who were on a FXai at the time of infection, such as patients with AFib. When performing these studies, it will be important to take into account the comorbidities of the patients and stratify the study groups accordingly to provide a true comparison of clinical outcomes. For example, it would be inappropriate to compare a 60-year-old patient on an FXai for atrial fibrillation (AFib) to a 90-year-old patient on FXai for AFib with concurrent congestive heart failure and/or chronic lung disease, as the latter will have a significantly higher chance of developing severe COVID-19. Additionally, due to COVID-19 testing limitations, it is likely that only the patients who develop symptoms will be captured in such a retrospective study and will miss those patients who may have been exposed and infected, but did not get ill enough to seek testing and/or medical care. This type of large dataset analysis could provide valuable information regarding whether coagulation status at the time of SARS-CoV-2 infection is a factor in the progression and severity of COVID-19 and whether FXai may be protective in asymptomatic and mild COVID-19 patients.

## Hypercoagulability and Inflammation Are Intimately Related to COVID-19 and the Link Is Not Limited to Factor X

While we focus on the potential role of FXa in COVID-19-related disease in this review, there are multiple other mechanisms of coagulation/inflammation activation, including platelet-mediated, complement-mediated, and endothelium-mediated pathways that should not be ignored. In fact, it is almost certain that there are various pathways activated simultaneously via different routes during different phases of COVID-19. While FXa inhibition may be found to be efficacious early on in the disease, it would not be surprising if patients with severe inflammation and derangement of their coagulation system, such as those requiring intensive care and/or mechanical ventilation, may benefit from anticoagulants that are further downstream of the FXa pathway, such as thrombin inhibition via UFH or a direct thrombin inhibitor. This is especially true in the cases such as widespread endothelium involvement with excess tissue factor expression and thrombin generation. In fact, the level of systemic inflammation and clinical status of the patient, such as whether admitted to the intensive care unit, may be one of the explanations for the variation in clinical reports on the frequency of thromboembolism and location of the thrombi. 116 117 118 Additionally, current reports and studies are very geographically diverse and describe observations in a wide variety of patient populations which likely impose additional important confounding variables, including differences in health care status, such as genetics, comorbidities, and socioeconomic status, that can affect the severity and progression of the disease as well as the ability to seek early treatment. 119

It is likely that a multimodal approach to coagulation management, exploring other factor inhibitors (factor XII or XI/XIa), platelet inhibitors, PAR inhibitors, and complement inhibitors (for which many drugs are currently under investigation), may be the most beneficial approach. Ideally, treatment would include a combination of anti-inflammatory and anticoagulant drugs along with an antiviral agent (if and when available). A close examination at the role of platelets in COVID-19 is also justified, considering the lack of consistent thrombocytopenia that is typically present in other systemic inflammatory conditions. 120 Just as control for pain and inflammation often requires a polypharmacy approach, such as the administration of opioid or local anesthetic in combination with nonsteroidal anti-inflammatory agents, management of complex coagulation derangement may also benefit from this type of approach, such as combining anticoagulation with anti-inflammatory agents, such as steroids, in patients with severe COVID-19. 121 New anticoagulants under development and in clinical trials, such as factor XI/XIa inhibitors, may prove to be beneficial as both anti-inflammatory and anticoagulant agents, similar to factor Xa inhibitors. 122 At this time, these drugs are not yet currently commercially available and, upon the involvement of the extrinsic coagulation pathway, such as with endothelial cell tissue factor expression, there may be a need for a common pathway coagulation inhibitor, such as a factor Xa or IIa inhibitor. In fact, there may be a strong case for the eventual use of factor XI/XII inhibitors and factor Xa/IIa inhibitors simultaneously, as both target different inflammation and coagulation activation pathways and may work synergistically, although dosing will need to be worked out so as to not result in overanticoagulation and adverse bleeding events. There is also the potential to explore the administration of FXai and other factor inhibitors via direct inhalation, as with the studies with UFH and tPA, for the treatment of respiratory distress and ARDS, although both dose and formulation would need to be optimized and tested.

While this article does focus on the potential role of FX in the propagation of inflammation and coagulation in the setting of COVID-19, it is important to keep in mind that the activation of the coagulation pathway does not always result in pathology, and is primarily meant to be protective against the consequences of infection and trauma. For example, FX/FXa has been shown to interact directly with PAR-2 and indirectly with endothelial protein C receptor–dependent recruitment of PAR-1 and appears to play a protective role. 123 As the pathophysiology of COVID-19 is better understood and more tailored therapeutic approaches to anticoagulation are evaluated, it will be essential to ensure that the protective role of the coagulation pathway is not completely eliminated and that we do not cause adverse bleeding events. Similar to the use of steroids for the treatment of infection-related inflammation, there are always two sides of the coin, where steroids can reduce inflammation while also inhibiting the body's ability to appropriately respond to and control the invading pathogen. 124

## Conclusion

In this review, we discuss the potential role of coagulation factor Xa in the mechanism of infection and pathophysiology of COVID-19. The potential role of FXa in COVID-19 illness is multimodal, including participating in SP cleavage and cell infection, and perpetuating immune cell activation, local and systemic inflammation and coagulation. Patients with preexisting conditions, including heart and lung disease, are at an increased risk for severe illness secondary to SARS-CoV-2 infection and it is known that FXa may play a role in both cardiac dysfunction and acute and chronic pulmonary inflammation and fibrosis. FX/FXa is also coexpressed in multiple cells that express ACE2, both of which have been shown to be increased in expression during various acute and chronic disease states. Based on this information, the administration of FXai may be a potential prophylactic and therapeutic treatment for COVID-19, resulting in the reduction of cell infectivity and, therefore, viral load, as well as the reduction in systemic inflammation and coagulation. Further studies, exploring the role of coagulation factors in COVID-19 and the potential of FXai as therapeutic agents, alone and in combination with other therapeutics, are warranted.
